# Assessing the sustainability of daily chlorhexidine bathing in the intensive care unit of a Veteran’s Hospital by examining nurses’ perspectives and experiences

**DOI:** 10.1186/s12879-017-2180-8

**Published:** 2017-01-14

**Authors:** Jackson S. Musuuza, Tonya J. Roberts, Pascale Carayon, Nasia Safdar

**Affiliations:** 1 William S. Middleton Memorial Veterans Affairs Hospital, Madison, WI USA; 2 School of Nursing, University of Wisconsin-Madison, Madison, WI USA; 3 Department of Industrial and Systems Engineering, University of Wisconsin-Madison, Madison, WI USA; 4 Department of Medicine, University of Wisconsin School of Medicine and Public Health, Madison, WI USA; 5 Center for Quality and Productivity Improvement, University of Wisconsin-Madison, Madison, WI USA

## Abstract

**Background:**

Daily bathing with chlorhexidine gluconate (CHG) of intensive care unit (ICU) patients has been shown to reduce healthcare-associated infections and colonization by multidrug resistant organisms. The objective of this project was to describe the process of daily CHG bathing and identify the barriers and facilitators that can influence its successful adoption and sustainability in an ICU of a Veterans Administration Hospital.

**Methods:**

We conducted 26 semi-structured interviews with a convenience sample of 4 nurse managers (NMs), 13 registered nurses (RNs) and 9 health care technicians (HCTs) working in the ICU. We used qualitative content analysis to code and analyze the data. Dedoose software was used to facilitate data management and coding. Trustworthiness and scientific integrity of the data were ensured by having two authors corroborate the coding process, conducting member checks and keeping an audit trail of all the decisions made.

**Results:**

Duration of the interviews was 15 to 39 min (average = 26 min). Five steps of bathing were identified: 1) decision to give a bath; 2) ability to give a bath; 3) decision about which soap to use; 4) delegation of a bath; and 5) getting assistance to do a bath. The bathing process resulted in one of the following three outcomes: 1) complete bath; 2) interrupted bath; and 3) bath not done. The outcome was influenced by a combination of barriers and facilitators at each step. Most barriers were related to perceived workload, patient factors, and scheduling. Facilitators were mainly organizational factors such as the policy of daily CHG bathing, the consistent supply of CHG soap, and support such as reminders to conduct CHG baths by nurse managers.

**Conclusions:**

Patient bathing in ICUs is a complex process that can be hindered and interrupted by numerous factors. The decision to use CHG soap for bathing was only one of 5 steps of bathing and was largely influenced by scheduling/workload and patient factors such as clinical stability, hypersensitivity to CHG, patient refusal, presence of IV lines and general hygiene. Interventions that address the organizational, provider, and patient barriers to bathing could improve adherence to a daily CHG bathing protocol.

**Electronic supplementary material:**

The online version of this article (doi:10.1186/s12879-017-2180-8) contains supplementary material, which is available to authorized users.

## Background

Healthcare-associated infections (HAIs) lead to increased morbidity, mortality and medical costs [1–3]. In the United States alone, about 722,000 people get an HAI every year and 75,000 people with HAIs die [2]. Zimlichman et al., considering only the five major HAIs, estimated that HAIs cost the United States healthcare system $9.8 billion annually [1]. Daily bathing with chlorhexidine gluconate (CHG) for intensive care unit (ICU) patients has been shown to reduce healthcare-associated bloodstream infections (BSIs) [4–11] and colonization by multidrug resistant organisms (MDROs) [5, 6, 10].

A lot of evidence about interventions to reduce HAIs has been generated in recent years. However, there is still a substantial gap between evidence and practice in the field of HAI prevention in general [12]. Therefore, in order to reduce the health and economic burden of HAIs, there is urgent need for the translation and sustainability of proven efficacious interventions into healthcare practice.

Implementation research is critically needed to facilitate translation of evidence into practice [13], and this research has not been done for daily CHG bathing. For an efficacious intervention such as CHG bathing, it is important to understand all the factors that can influence its successful adoption and sustainability. Sustainability generally refers to the continuation of an intervention or its effects [14, 15]. It is an essential consideration in HAI prevention interventions in order to maintain the initial momentum that occurs when the intervention first gets implemented. The long-term viability of an HAI prevention intervention is important because the hospital leadership will allocate scarce resources to efficacious and successful interventions [15, 16]. Crucial factors that influence sustainability of health care interventions include 1) factors in the broader environment; 2) those within the organizational setting; and 3) project design and implementation factors [14].

Sustainability of an intervention can be assessed in various ways such as 1) examining whether its pertinent activities and resources continue to support its primary objectives [17]; 2) examining whether there is continuation of its implementation strategy [18]; and 3) examining whether it is accepted in the institution particularly by those who actually carry it out [19, 20]. Since daily CHG bathing is a nursing task, understanding nursing staff’s perspectives and experiences with CHG bathing is key to understanding the factors that impact its sustainability.

As part of a quality improvement project to assess compliance to daily CHG bathing, we conducted direct observations of the bathing process, gathered data on CHG usage, and examined electronic medical records (EMR) for documentation of CHG bathing. After observing lower than expected compliance to daily CHG bathing (results not shown in this paper), we embarked on a qualitative inquiry to find out factors that might explain results from this prior project.

The objective of this project was to describe the process of daily CHG bathing in the ICU of a Veterans hospital from the perspective of nursing staff, and identify factors that impact its adoption and sustainability. In addition, we specifically asked about participants’ views about adding daily CHG bathing to the patient’s order set as an intervention to improve compliance to CHG bathing by nurses.

## Methods

### Overview and design of the project

#### Setting and participants

This study was conducted in the ICU of a 129-bed Veterans Hospital (VA) in Wisconsin, USA. This hospital provides tertiary medical, surgical, neurological, and psychiatric care, and a full range of outpatient services. At the time of this project, it had two ICUs—the medical-surgical ICU with 7 beds and the cardiac ICU with 6 beds.

This VA facility implemented daily CHG bathing in the ICUs in May of 2014 and uses Hibiclens^®^ soap (Hibiclens^®^ 4%). Training on CHG bathing was provided during four weekly staff meetings one month prior to switching from regular soap to CHG soap. During training, staff were informed about the steps involved in the process of CHG bathing and situations when CHG bathing would be contraindicated. Staff were also provided with written material covering various topics about CHG bathing and CHG frequently asked questions. The bathing process involved several steps starting from gathering the needed supplies to application of a lotion to keep the patients skin moisturized after the bath. Staff were required to document completion of the bath in the EMR.

Participants in this project included nurse managers (NMs), registered nurses (RNs) and health care technicians (HCTs) or certified nursing assistants (CNAs) working in the ICU. In this paper we use the term HCTs rather than CNAs, and also, for clarity, RNs and HCTs are referred to as nursing staff.

#### Study procedures and data collection

Nurse Managers introduced the first author to the unit and granted him permission to access the unit. The aims of the project were presented to the staff who were invited to participate. Interviews were scheduled with all willing nursing staff. We used an interview guide (appendix) and conducted semi-structured interviews in a quiet room on the unit.

Questions in the interview guide were broadly framed using the Systems Engineering Initiative for Patient Safety (SEIPS) model as the main framework. The SEIPS model is a sociotechnical systems approach that can be used to effectively address contextual factors necessary for the successful design and implementation of an intervention [21, 22]. It focuses on five interacting elements of the work system— person, tasks, tools and technologies, physical environment, and organizational factors. Interactions of these elements can affect care processes (e.g. patient bathing), which result in patient outcomes such as quality of care and patient safety, and organizational outcomes such as efficiency and acceptance of interventions. The five work system elements served as topics in the interview guide. This enabled us to ask about who was involved [Person]; what they did [Tasks]; the kind of tools/technologies they used [Tools/technologies]; issues related to patient rooms and the unit in general [Environment]; and organizational factors, for example leadership that influences the CHG bathing process [Organization]. Examples of questions (related to organization) were: 1) “How do you communicate with the other nursing staff that a chlorhexidine bath for a given patient was done?” and 2) “Please tell me what you know about the chlorhexidine bathing policy.” The SEIPS model was appropriate for this project because it informs contextual factors that impact implementation and sustainability of interventions [21].

Based on our previous work examining CHG bathing practices in this hospital, we hypothesized that an intervention that involved adding daily CHG bathing to the patient’s order set might increase compliance to CHG bathing. Consequently, we specifically asked participants for their views about such an intervention.

Convenience sampling was used for the recruitment of participants; almost all the nursing staff on the units willingly participated in the interviews. We mostly conducted individual interviews, but on four occasions nursing staff were interviewed in groups of 2–3, particularly at times when the unit was not very busy. A total of 26 individuals were interviewed: 4 NMs, 13 RNs and 9 HCTs. Four interviews occurred in groups as follows: 3 RNs; 2 HCTs; 1 HCT and 1 RN; and 2 RNs. The duration of the interviews ranged from 15 to 39 min, with group interviews taking slightly longer time than individual interviews.

The interviewer (JSM) regularly met with two co-authors (TR and NS) to discuss progress of the interviews. During these meetings the authors analyzed emerging themes and brainstormed about interview questions that would capture best the depth of these themes. We asked probing questions to expand on participants’ responses and to increase on the depth of the interviews. An example of a probe used was: “You mentioned that you are motivated to conduct CHG baths because CHG bathing is hospital policy, tell me more about that.”

#### Ethical considerations

The University of Wisconsin Minimal Risk Institutional Review Board exempted this project as it was qualified as quality improvement. All participants were informed about the purpose of the project and voluntarily participated. We obtained permission to audio record interviews; participants were assured that their responses would be kept confidential and that they would not be identified individually in any reports or publications. Those who agreed to participate signed a document indicating their agreement to voluntary participation.

#### Data analysis

Professionals from a registered transcription company transcribed all interviews verbatim. We used qualitative content analysis to code and analyze the data [23, 24]. To start, the first author read the transcripts several times to get familiar with the data and noted down initial ideas. This was followed by line-by-line coding in which the text was divided into meaningful units (words, phrases, sentences, or sections), which were labeled with relevant codes. We coded for patterns within the data, including frequency (how often concepts appeared), sequence (the order in which they appeared), correspondence (how they occurred in relation to certain activities), similarity (whether the concepts were happening the same way), difference (how different they were) and causation (if they appeared to lead to another) [25]. The first author regularly met with the co-author (TR) to review the coding process. The next step was to collect related sub-categories into categories or themes. Defining each category or theme and its specifics was an ongoing process. To organize, sort, and code the data, interviews were imported into Dedoose, Version 6.1.18. Los Angeles, CA [26].

#### Rigor of data analysis

Two authors (JSM and TR) met regularly to review and discuss the coding process. Lack of clarity on how to code a certain section of the data was resolved through a discussion until consensus was reached. To further ensure trustworthiness of the interview data analysis and, therefore, scientific integrity, we conducted member checks. We returned a summary of our interview findings to a small number of participants to confirm that we were correctly representing their perspectives. We kept an audit trail of all the decisions made during the iterative process of collecting and analyzing data [27, 28].

## Results

**Fig. 1:**
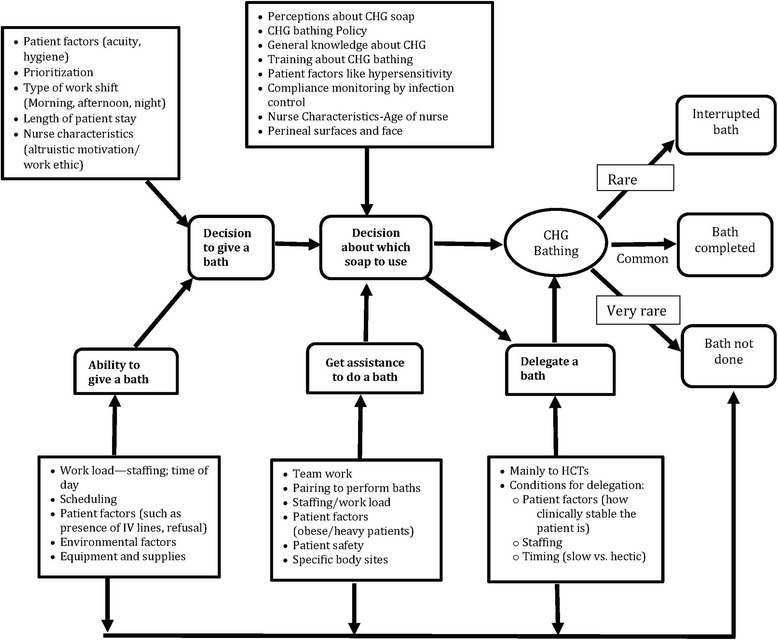
Interrelationships between different conditions needed for completion of a chlorhexidine bath. Legend: the direction of the arrows means that factor (s) in the text box from which the arrow starts influence factors in the box into which the arrow points. Bolded text indicates the five steps of patient bathing

We identified five steps of bathing described by participants: 1) decision to give a bath; 2) ability to give a bath; 3) get assistance to do a bath; 4) delegation of a bath; and 5) decision about which soap to use. The bathing process resulted in one of the following three outcomes: 1) complete bath; 2) interrupted bath; and 3) bath not done. Interactions between the five bathing steps and the resulting three CHG bathing outcomes are summarized in Fig. 1.

Generally, participants did not make a distinction between CHG bathing as an infection prevention procedure and CHG being another kind of soap that could be used for patient bathing. Therefore, choosing to use CHG soap was just one of the steps involved in patient bathing.

Verbatim illustrative quotations from the interviews (Q) within the five bathing steps are presented in Table 1 (online Additional file 1: Table S1).

**Table 1:** Patterns coded within the data and examples of associated quotations and codes

Pattern within the data	Notes and quotations	Code (s)
Frequency	This relates to how often concepts appeared in the data. As an example, the code “perception about CHG soap” had the highest frequency, being coded 46 times in all the interviews combined. Therefore, frequency was one way that informed the discussion and conclusions about the importance or significance of perceptions about CHG. Below are examples of quotations associated with this code: *Quotation 1:* “*Yeah*, *I mean it gives you justification for why you are doing it because sometimes it is nice to say this is why we do it not just you have got to do it. I think people understand why and the importance there and do it more.”* *Quotation 2*: “*Because I think it*, *I just don*’*t think it*’*s*, *I look more from a skin standpoint and how it*’*s affecting the patients and their skin and how dry it*’*s getting.”*	• Perception about CHG soap
Sequence	This refers to the order in which concepts appeared. For example, when participants described the sequence of conducting a bath and the different steps involved as shown in the quotation below: “*So like from start to finish*, *from grabbing the supplies*, *to getting your help*, *conduct the bath and then documenting it*.”	• Gathering supplies • Getting help to do a bath • Conduct and document bath
Correspondence	This refers to how concepts occurred in relation to certain activities. For example, activities such as changing the patient’s linen which might have happened during the bathing process and potentially interrupted or prolonged the time of the bath and necessitated the need for assistance. “…*depending on the patient*, *if I need*, *I can start a bath by myself*, *but if I need someone to help me roll them to get his back and change the linen*, *then I get help*.”	• Getting help to do a bath
Similarity	This refers to whether the concepts were happening the same way or had the same meaning. For example, two participants could have meant or implied the same idea using different sentences. These two quotations relate to workload. *Quotation 1*: “*Techs call off a lot. If we don*'*t have a health tech*, *then I will do as much as I can*, *and then when it comes to the turning part I*'*ll ask for help. And if*, *depending on how the staffing is*, *so staffing could be an issue. If it*'*s really busy*, *and there*'*s like say*, *for example*, *we usually*, *minimal staff for us is three…*” *Quotation 2*: “*And sometimes our techs are pulled if there*'*s a sitter need on a floor. Sometimes they need sitters*, *and a lot of times when it happens they look to us for our sitter or for our health tech. You know*, *we might give her up for two*, *three hours. They might have a huge need*, *and they can*'*t get anybody. So they*'*ll say*, *well*, *we have to take her because it*'*s a suicide watch or whatever*, *and they have to have somebody. So then*, *again*, *we*'*re left without a health tech*.”	• Heavy workload and staffing shortage
Difference	This relates to how different concepts were. For example, quotation 1 below refers to clinical stability of a patient while quotation 2 refers to environmental factors affecting CHG bathing.	
	*Quotation 1*: “*A lot of it depends on a patient. Because if a patient is a really stable*, *our health techs can do that*, *but if they*’*re not*, *if it*’*s an unstable patient*, *then it*’*s appropriate for the nurse to be involved*.”	• Clinical stability
	*Quotation 2*: “*The rooms are small with a lot machines and some do not have warm water in the sinks*.”	• Environmental factors
Causation	If concepts appeared to lead to another or to one of the outcomes (completed bath, interrupted bath and bath not done). For example, in the quotation below, getting assistance leads to a faster bath which reduces the likelihood of patient refusal of baths. “… *because if you have two people on staff and could do the bath*, *one person can do the cleaning and then one person can do the rinsing. And then the timeframe for the bath can be shorter if you have someone good that you work with. The patient will not refuse the next bath if the previous is done fast*.”	• Patient refusal • Short bath duration • Getting help to do a bath

### Decision to give a bath

The first step in the CHG bathing process was deciding to give a bath. This step was influenced by the purpose of, and the priority for giving a bath. Participants described a number of reasons why bathing was important. They believed bathing was a fundamental nursing job duty that primarily provided patients with comfort and attended to patient personal hygiene and dignity. In addition, bathing served a functional purpose for nurses; it provided them an opportunity to perform thorough skin assessments, prompting identification of actual and potential skin issues. Participants also described in some cases that bathing was important for infection prevention (Q1).

The priority for a bath was related to its purpose and its fit with other patient needs, timing, and organization of activities on the unit. Because bathing was perceived as a comfort measure amidst the many other activities nursing staff carry out, giving a bath was a low-priority activity (Q2). Patient acuity influenced the level of nursing care required, which in turn influenced the nursing staff’s decision to carry out a bath. If a patient had other urgent needs, then a bath was delayed. Examples of these needs included preparation for tests or procedures, need for critical, important or time-sensitive medications, and continual or frequent monitoring of hemodynamic stability. In some cases, patients were described as clinically unstable during the bath and some refused baths midway, particularly when they were taking long. In these cases, baths would get interrupted before completion. Furthermore, a number of potentially competing patient needs made bathing a lower priority, such as activities of daily living and the need for ambulation (Q3). The decision to give a bath was also influenced by how long a patient had been on the unit and how often they had received baths during their stay. Patients who had stayed longer on the unit before getting a bath would receive baths before those who had just been admitted to the unit (Q4). Some participants stated that patients’ baths did not have to be given right after admission implying that they could wait for up to 24 h, based on unit CHG bathing policies, before a bath was required following admission (Q5).

Participants also described how the unit had some particularly busy times, which lowered the priority for bathing during these situations. Some participants saw giving a bath as taking a lot of time away from other patient activities, particularly when giving baths to immobile patients who could not participate in their bath (Q6). The morning shift involves many activities such as taking patients for procedures, and performing ADLs. Participants often reported making a decision to defer bathing to the later shifts to accommodate all of these activities during the morning shift. Participants reported that, generally, baths were easier to give during the afternoon shift or the evening shift (Q7).

There were some cases where participants described making bathing a priority. In these cases, baths would get completed. When bathing was believed necessary for infection control, it was assigned higher priority (Q1). In addition, bathing was given higher priority for patients with poor personal hygiene (Q8). Nurses assess the patient’s general hygiene when they first come to the unit. The assessment focuses primarily on the cleanliness and grooming of the external body including body odor, but they also look out for hair, oral, nail and wound hygiene if relevant.

Finally, communication between nursing staff about CHG bathing was another factor that influenced the decision to give a bath. Nursing staff communicated to each other about CHG baths in three ways: 1) verbally during the hand-off report; 2) documenting a completed or needed bath on a white board located by the nurse station on the unit; and 3) documenting in the EMR. CHG bathing was not always noted on the board or not always talked about during the hand-off report (Q9 and Q10). Given how fast paced the units were, it was difficult for nurses to always check completion of a CHG bath in the EMR. If they could not easily tell whether a bath was given, nurses made the assumption that it was done for that day.

### Ability to give a bath

Participants’ ability to give a bath was influenced by workload (staffing and time of the day), scheduling issues, environmental factors and organizational or administrative support.

Participants reported that staffing shortage and high-pressure ICU work conditions, which involve taking care of critically ill patients, hindered their ability to conduct baths. Staffing shortage was described in two ways: 1) insufficient staff scheduled to work, 2) redistribution of scheduled staff to other units. Insufficient staff scheduled to work occurred particularly during academic periods when students take classes; most of the HCTs are enrolled in nursing school and their hours are limited during school time. Moreover, we learned from Nurse Managers and from earlier direct observations that baths were mainly done by HCTs rather than RNs. Sometimes HCTs were called to help on other units and, therefore, could not perform their ICU duties (Q11). Nurses also stated that, many times, HCTs did not report for work and there was no replacement for them. Absence of floating HCTs made it difficult for baths to get done when HCTs were not available (Q12).

Participants described several patient factors that also affected their ability to perform baths. Some patients refused to have baths for reasons such as dislike of the smell of soaps used or they simply preferred to rest rather than have a bath. How a bath was communicated to the patients influenced whether they refused to have a bath. Informing the patients that they will be getting a bath was associated with higher acceptance than asking patients if they would like to have a bath (Q13). Participants also stated that it was difficult to give a bath to obese patients or patients with intravenous lines. Also, some patients were independent and chose to do their own baths.

Difficulty to schedule baths was another hindrance to conducting baths reported by the nursing staff. There were no set scheduled times for conducting baths. Consequently, baths were conducted whenever it was convenient for the staff. Although baths were supposed to be given during one of three shifts— morning (AM) shift, afternoon (PM) shift and night shift, most of the baths were done during the PM and night shifts when the unit was less busy; the baths could also be coupled with other patient evaluations (Q14). Given these staffing and scheduling challenges, nursing staff sometimes conducted baths in situations where they were absolutely needed, for example, when patients were heavily soiled (Q15).

Environmental factors such as small ICU rooms with clutter made it difficult for nursing staff to conduct a CHG bath. An additional inconvenience was the need to get water from sinks located outside the patients’ rooms when the sinks in the rooms did not have warm water (Q16).

Despite the challenges in the ability to give a bath, participants stated that they always had the needed equipment and supplies; for example, they never ran out CHG soap; it was well stocked wherever and whenever they needed it.

### Get assistance to do a bath

Participants described a number of situations in which they needed the assistance of coworkers to complete a bath. Assistance was generally needed for the patient’s backside, for immobile or heavier patients who needed to be moved or rolled to their side, for rinsing after applying soap and for changing linen. When it was available, assistance made baths go faster, which reduced patient refusal of baths because patients were not left alone cold and they were much more comfortable (Q17). When baths were conducted in pairs, patient safety was potentially enhanced; with the extra person, patient falls particularly when patients had to be turned were less likely to occur.

Participants stated that that needed assistance was not always available (Q18), particularly during hectic times like the morning shift when staff were heavily engaged in other patient care activities (Q7). During such times, baths were rarely conducted.

### Delegation of a bath

Baths would sometimes get delegated for a number of reasons. Participants reported that the delegation was usually from RN to HCT, but could also happen from RN to RN or HCT to HCT. RNs mostly delegated baths for clinically stable patients to HCTs (Q19). Delegation of baths occurred during hectic or busy times when a lot of other activities were taking place on the unit. When delegation occurred, it would increase the likelihood that a bath would get done.

Nonetheless, participants felt that delegation did not always happen when it should and this hampered the CHG bathing process generally. For unclear reasons, probably related to perceived importance of baths, sometimes RNs did not delegate baths to HCTs. When baths were not delegated, they would not get done (Q20).

### Decision about which soap to use

Participants could choose between using ordinary soap and using CHG soap to conduct baths. The choice between these two kinds of soap depended mainly on knowledge, attitudes, beliefs and perceptions about the CHG soap.

Participants had different views about CHG soap. Some believed that it is valuable in preventing hospital-acquired infections (Q1) and therefore used CHG soap in order to make this benefit available to their patients. Others believed that CHG soap destroys the normal microbial flora and instead increases the patients risk to infection (Q21). They believed that CHG has certain characteristics such as being a harsh soap, which makes it unsuitable for older patients whose skins are already frail. The daily use of CHG was also of concern to participants because they believed that this was too frequent and that this constant exposure of already frail skins to a harsh soap such as CHG was detrimental for their patients (Q22). Others thought that because CHG soap is not used post discharge from the ICU, they did not see the point of using it while in the ICU. They also stated that their facility has very low HAI rates as compared to many other facilities, so they did not see the need for extra infection prevention measures like daily CHG bathing.

Certain nurse characteristics influenced the choice between ordinary soap and CHG soap. Older nurses tended to prefer ordinary soap and water than CHG soap. They believed that CHG soap did not fully clean the patient as ordinary soap and water does (Q23).

Hypersensitivity to CHG, a patient factor, also influenced the decision about which soap to use. Nursing staff learned about a patient’s hypersensitivity from the EMR or from the patients. In cases of hypersensitivity, CHG soap was avoided and another kind of soap was used. Participants believed that the patients in their facility (VA hospital) are much older and therefore have frail skins that should not be subjected to a harsh soap such as CHG. Some insisted that they needed to see studies that demonstrated CHG’s efficacy replicated in the VA population (Q24).

Some participants chose CHG baths only because it is hospital policy. Therefore, they used it not necessarily because they believed that it is beneficial or special in any way. This general lack of buy-in was because some participants did not believe that CHG soap was different from ordinary soap. In fact, they believed that apart from cleaning patients better than CHG soap, ordinary soap does not leave the patients’ skin dried up (Q22).

General knowledge about CHG and training about CHG bathing was another factor that influenced the choice of soap to use. When daily CHG bathing was first introduced, participants received in-service training about it through staff meetings and electronic mails (e-mail), and were also provided with a document (protocol) to read about the procedure. They thought that the training was not very well structured and CHG bathing was not practically demonstrated to them. They also reported that refresher training about the procedure is rarely done and that no formal training is given to newly hired staff members. This perceived inadequate training did not seem to have affected their skills in performing the bath per se, but rather seemed to have made them believe that leadership did not consider CHG bathing a very important intervention. For example, some participants expressed concern that the general compliance to daily CHG bathing by the staff on a unit was monitored by the infection control (IC) department rather than by the unit managers. They believed that this takes away the ownership of the procedure from the unit managers.

### Adding CHG bathing to the order set

As noted in the methods section, we specifically asked participants for their views about an intervention that involved adding CHG bathing to a patient’s order set. Some participants felt that this might compel them to give the CHG baths and hence increase compliance to daily CHG bathing (Q25). However, some were against it and mentioned that it would clutter the already crowded order sets. They also felt that it might blur the nurse and physician responsibilities, since bathing is essentially a nursing procedure and should be left entirely to nurses. They also felt that it might not actually improve compliance since staff might simply scan the CHG soap without actually using it. They also felt that this intervention might not be feasible to implement and that patient refusal will imply refusal of a treatment which has its own ramifications.

## Discussion

This quality improvement project was designed to describe the process of daily CHG bathing and to identify challenges in the implementation and sustainability of the intervention in an ICU setting. However, participants did not make a clear distinction between CHG bathing and bathing in general. They believed that CHG soap was just another kind of soap available for patient bathing. Choosing to use CHG soap was only one step of several steps necessary for conducting a patient’s bath. Therefore, our project described factors that influence patient bathing in general many of which ultimately influence CHG bathing since it is one of the steps in patient bathing.

Understanding that nurses do not make a distinction between CHG and ordinary soap bathing is important. On one hand, a hospital unit could choose to stock only the CHG soap and then the nursing staff will have no other choice but to use it; however, this may not be practical because it would mean that there would be no alternatives in case of patient sensitivity or allergy to CHG. Also, within each bath, some body parts such as the face and perineum should not have soap applied to them. More studies need to examine if using only a wet wash cloth without soap on these body parts is safe for patients. A lack of distinction between bathing patients with CHG versus regular soap is in contrast with the findings of a previous quality improvement project by Hines et al. [29], which showed that most nurses and patient care technician (PCTs) were aware that for inpatient bathing, CHG should be used instead of regular soap. This implies that they made a distinction between CHG bathing and bathing in general using regular soap. Eigsti [30], showed that 68% of nurses preferred using CHG over regular soap that is used for general bathing. Participants in Eigsti’s study also made a distinction between CHG bathing and general bathing with regular soap. It is important to note that these studies asked about participants’ preference and awareness/knowledge of whether CHG should be used rather than regular soap. Such questions were more likely to elicit socially desirable answers than in our project where we asked about nurses’ perception of the soaps. Unlike our project that employed semi-structured interviews, both of these studies used surveys that might not have provided an opportunity to obtain a richer and deeper understanding of the nurses’ perspectives on patient bathing.

In our project, one of the main barriers to bathing was perceiving bathing as a comfort measure and therefore giving it a low priority amidst the many other activities nursing staff carry out. An ICU is a fast-paced high-stress environment, therefore prioritization is very crucial in this setting [31, 32]. However, beyond patients’ comfort, bathing of ICU patients has other benefits such as patients’ relaxation, reducing pyrexia and stimulating circulation [33]. In order to mitigate the perception of low priority given to bathing, it is crucial that these other clinical benefits of patient bathing get emphasized to healthcare workers (HCWs) conducting patient baths. For CHG bathing in particular, there is need for more education of HCWs conducting baths about the importance of CHG bathing as an infection prevention intervention. Formal short training sessions focusing on CHG bathing could be used to emphasize the importance of this intervention.

Heavy workload and staffing shortage were other barriers to conducting baths. Heavy workload concerns in the ICU are well known and evidence indicates that nurse burnout is a common problem in the ICU [34, 35]. With burnout due to heavy workload, it is difficult for staff to accomplish many of their tasks, patient bathing being one of them. Also, compared to other general care areas, ICUs are affected by high vacancy rates and turnover [32]. High turnover would imply greater need for frequent training when new staff comes in and staff that are already trained in patient bathing leave. Participants in this project suggested that scheduling baths could indirectly help alleviate the heavy workload; baths could be scheduled during less busy shifts. For example, patients in odd rooms could get their baths during the day shift and others later on or a certain number of baths could be given per shift depending on how many staff are available on that shift. Although this suggestion is intended to only impact the scheduling of patients baths, it is difficult to implement given the complexity of bathing and all the various factors that influence it, and do not necessarily systematically align with room numbers.

Another important finding specific to CHG bathing was that some nursing staff seemed to imply that when a patient gets admitted to the unit, they have a 24-h window before they can actually start adminstering baths to them. This perception of having a 24-h window before nurses could start the bathing schedule for a particular patient can potentially lead to missed patient baths. This idea could have resulted from a misunderstanding of the hospital’s ICU CHG bathing protocol, which requires a single bath every 24 h. This is another opportunity for staff education on the CHG bathing protocol.

Communication about CHG baths in particular was one of the factors that influenced the choice of soap by nursing staff. Communication may break down particularly during handoffs and could result in undone patient baths. Breakdowns in communication during handoffs have been extensively documented in literature and are associated with poor patient outcomes [36]. Some of the strategies that have been tried include education for HCWs to perform effective handoffs [37] and the use of tools such as online forms and checklists [38]. In this project, participants suggested that staff should be encouraged to communicate about CHG baths by word of mouth at handoff.

In this project we also learned that communication about CHG baths had a significant impact on patient refusal, one of the barriers to actually conducting patient baths. Informing patients that they would be getting a bath was associated with much higher acceptance than asking patients if they liked to have a bath. A strategy such as standardized bathing communication messages could help to empower HCWs to effectively communicate information about baths to patients. Hines et al., reported that patient refusal was one of two major barriers to daily bathing of patients [29]. Providing educational support about the importance of CHG bathing to patients and their families was the most common suggestion to improve patient compliance to CHG bathing [29].

One of the interventions to improve compliance to CHG bathing suggested by participants was to include CHG bathing in the patient’s order set and to administer it as a bar coded medication. Bar coded medication administration systems (BCMAs) have been shown to reduce medication errors [39, 40]. Some participants in this project thought that implementation of BCMAs for CHG might increase nurses’ compliance to CHG bathing. Others thought that it might not increase actual baths conducted because there are always workarounds, where nurses might scan the CHG soap but not actually conduct the bath. Workarounds in the use of BCMAs have been extensively described [41]. To the best of our knowledge, our project was the first to seek nursing staff opinions about BCMAs use for CHG bathing. Future studies need to further explore this by seeking the perspectives of other healthcare team members, such as physicians.

We also found that for unclear reasons, probably related to the perceived importance of baths, sometimes RNs did not delegate baths to HCTs; when baths were not delegated, they were not done. This is an area that needs further examination in future studies.

Participants in this project suggested that the use of CHG-impregnated wipes as opposed to the CHG foam soap could increase compliance to CHG bathing. This was because of the perception that baths given with CHG wipes take a shorter time than those with CHG foam soap. This a plausible reason because unlike using CHG soap, there is no extra rinsing step when wipes are used [42]. Participants suggested that newly employed nursing staff need to be formally trained about CHG bathing rather than informally learning about it from their colleagues on the unit. The formal training could probably increase the priority nursing staff place on CHG bathing. Noteworthy is the participants’ mention that baths need to always be conducted in pairs. This would increase efficiency or speed up baths, something that might help reduce patient refusal of baths, particularly subsequent baths after the first one.

A limitation of this project is that the project was conducted at a single-center Veterans Affairs hospital setting; therefore, it is difficult to generalize the results to the general population of hospitals in the United States. Nonetheless, results may be transferable to other ICU settings. The other limitation is that we did not analyze the data according to professional titles—by separating findings of HCTs/CNAs, RNs or NMs. It is true that more HCTs/CNAs conduct baths than RNs, but they both spend considerable amounts of time on the units and so we did not expect that their experiences with patient bathing would vary a lot. Another possible limitation is that there might be bias introduced due to convenience sampling. We do not think this was a major limitation of this study because we interviewed more than 95% of the nursing staff on the unit. Therefore, results presented are representative of all the nursing staff on the unit.

In this project we made a plausible assumption that by studying and describing the process of bathing, we will be able to identify factors that affect adoption and sustainability of CHG bathing. For example: 1) If staff who conduct baths understand the clinical benefits of CHG bathing on top of other general bathing benefits, the assumption is that they will likely adopt the new process. Evidence shows that provision of knowledge to nurses and implementing evidenced-based interventions can improve quality care and patient outcomes [43, 44]. Any lack of knowledge or information identified by assessing the bathing process could be addressed by providing specific education. 2). Participants in this project mentioned that monitoring of CHG bathing should be a joint venture between the infection control department and the unit managers. They believed that this would increase buy-in and ownership of unit managers. We were able to identify this finding by assessing the entire bathing process. Moreover, senior staff buy-in and support is very important for the sustainability of interventions [45, 46].

## Conclusion

In conclusion, findings showed that patient bathing is a complex process affected by many factors: 1) patient factors such as clinical stability, hypersensitivity to CHG, refusal, presence of IV lines, general hygiene and obesity; 2) nursing staff specific factors such as nurse characteristics, nursing staff perceptions and beliefs about the value bathing and prioritization of baths; and 3) organizational factors such as staffing and heavy workload, scheduling and the capacity to delegate baths. Factors that specifically facilitated daily CHG bathing were mainly organizational and included the policy of daily CHG bathing, an unfailing supply of CHG soap, and support such as reminders to conduct CHG baths by nurse managers. Since CHG bathing was not perceived as different from the usual soap and water patient bathing, interventions that address the organizational, nursing, and patient barriers to bathing in general could improve adherence to a daily CHG bathing protocol specifically and also ensure sustainability of this intervention. Therefore, future interventions aimed at improving CHG bathing should focus on improving conditions necessary to give a bath in general.

## Additional file

Additional file 1: Table S1. Illustrative quotations from the interviews arranged within the five bathing steps and the order set intervention. (DOCX 49 kb)

## Abbreviations

BSIs: Bloodstream infections
CHG: Chlorhexidine gluconate
EMR: Electronic medical records.
HAIs: Healthcare-associated infections
HCTs: Health care technicians
HCWs: Healthcare workers
ICU: Intensive care unit
IV: Intravenous line
MDROs: Multidrug resistant organisms
NMs: Nurse managers
RNs: Registered nurses
SEIPS: Systems Engineering Initiative for Patient Safety
