# Supplementary material for: Assessing the sustainability of daily chlorhexidine bathing in the intensive care unit of a Veteran’s Hospital by examining nurses’ perspectives and experiences
Source: BMC Infect Dis. 2017 Jan 14;17:75. doi: 10.1186/s12879-017-2180-8 (PMC5237510; doi:10.1186/s12879-017-2180-8)
Supplement: Additional file 1: Table S1. — Illustrative quotations from the interviews arranged within the five bathing steps and the order set intervention. (DOCX 49 kb) [file 12879_2017_2180_MOESM1_ESM.docx]

**Table 1.Illustrative quotations from the interviews arranged within the five bathing steps and the order set intervention**

| Bathing step | Illustrative quotations |
| --- | --- |
| Decision to give a bath |  |
|  | Q1:"I think it’s really important, just because we are the surgical ICU, so a lot of these patients have incisions and, you know, they’re open to bacteria. So it’s super important to keep those areas clean.” |
|  | Q2: “You know, we don't think of baths as a medical issue, although, it is an infection control issue, so it should be treated that way. But, again, when we have a list a mile long of things that we need to do, and we're running from room to room assisting nurses and patients, then baths kind of go by the wayside, because that's something that's seen as dispensable. So on my hierarchy it's like airway, you know. I need them alive, so if I don't, if they don't get their bath, nobody's going to die from not getting their bath today.” |
|  | Q3:"It doesn’t pay to bathe someone who’s not breathing. You need them breathing, you need his heart beating. You need some of these things. How unstable is he? The more stable the patient is, the more of a priority it is.” |
|  | Q4: “For me, it depends on how long they’ve been here and when last they got a bath. So, if they’ve been there for a long time and they didn’t get a bath, usually I make them my priority. And also when they smell really bad, I make them my priority. I think technically you have 24 hours before you need do a chlorhexidine bath, so they just wipe them down with warm wipes.” |
|  | Q5: “" I think technically you have 24 hours before you need do a chlorhexidine bath, so they just wipe them down with warm wipes” |
|  | Q6: “So it’s not necessarily the bath itself and using the chlorhexidine that is a problem or time consuming. It’s the entire process. I mean, you can, even from a health tech standpoint, you can go into a room to give a bath and you’re thinking, I mean, a bath for a patient who is immobile and can’t help you, I mean, you’re looking at ten minutes maybe start to finish. And this stuff turns into like a half hour. And again, that, it depends on the senses and the acuity of our patients. |
|  | Q7: “So a.m. times are a little too busy to do it. With the p.m. shifts and stuff like that, we try to make sure that during the day a lot of things are done for the patient so that on the p.m.’s they have more time to provide that level of care. |
|  | Q8: “… usually when they don’t smell very nice or if they have the orange ChloraPrep [Skin Prep for Surgery] all over them, and I just want to get rid of that, then I'll ask them if they want a bath Sometimes they do, sometimes they don’t. |
|  | Q9: “I mean, we’re supposed to chart it in PICIS (EMR system for the VA), but half the time it’s in a memo and we don’t actually go back and look for it.”  “So I usually like to ask, because sometimes CHG bathing is not even brought up during the hand-off report, it’s not mentioned. Like this morning it wasn’t, and I said, do you know who’s had a bath and who hasn’t? So…” |
|  | Q10: “Yeah. It’s like sometimes it’s up on the board, sometimes they tell you in the hand-off report, sometimes they don’t, sometimes it’s charted. Then when patients say, tell you, oh, I had one for sure. And then you look it up and it’s not in there. It’s like, okay, well, are you lying, or was it not charted? So” |
| Ability to give a bath |  |
|  | Q11: “And sometimes our techs are pulled if there's a sitter need on a floor. Sometimes they need sitters, and a lot of times when it happens they look to us for our sitter or for our health tech. You know, we might give her up for two, three hours. They might have a huge need, and they can't get anybody. So they'll say, well, we have to take her because it's a suicide watch or whatever, and they have to have somebody. So then, again, we're left without a health tech.” |
|  | Q12: “Techs call off a lot. If we don't have a health tech, then I will do as much as I can, and then when it comes to the turning part I'll ask for help. And if, depending on how the staffing is, so staffing could be an issue. If it's really busy, and there's like say, for example, we usually, minimal staff for us is three. If we have four people we can usually help each other. Sometimes with three it can get kind of busy. I won't, a lot of times depending on how it looks, I won't even schedule a bath. So that might be a limiting factors, no health tech, three nurses, and it's kind of busy. Either A, I'm busy, or they're busy. That might be, I may say to myself, nay, this guy's not going to get it, you know, at least not on my shift, he might not get a bath.” |
|  | Q13: “Patients refuse a lot. Only I think there’s a way that you can ask them. If you go in there and say, hey, like I was wondering if you wanted to have a bath now. A lot of times they’re going to say, hmm, no, let’s wait until tomorrow. But if you say, hey, is now a good time for a bath or would you like to do it later after dinner? So you’re just kind of giving them a choice, now or later. Then they’re more likely to do it. So I think that’s kind of a big thing.” |
|  | Q14: “So the p.m.’s usually try to schedule baths with their evaluation. So if they have to do a 9:00 assessment it’s great to do a full bath, so you can do a full assessment with the patient at that time.” |
|  | Q15: “A lot of times in the ICU it’s as needed. If you have somebody that’s soiling a lot, a bath is more than needed, it’s just kind of as needed, per se.” |
|  | Q16: “The rooms are small with a lot machines and some don not have warm water in the sinks.” |
| Get assistance to do a bath |  |
|  | Q17: “I would say yes, because if you have two people on staff and could do the bath, one person can do the cleaning and then one person can do the rinsing. And then the timeframe for the bath can be shorter if you have someone good that you work with.” |
|  | Q18: “So I think that everybody needs to take responsibility for it. And in a perfect world, the nurse and the aide would do it together and that would just make sure that everybody got it done. If each nurse said, I have to get this done if it hasn’t been done already, then…” |
| Delegation of a bath |  |
|  | Q19: “A lot of it depends on a patient. Because if a patient is a really stable, our health techs can do that, but if they’re not, if it’s an unstable patient, then it’s appropriate for the nurse to be involved.” |
|  | Q20: “For a lot of reasons, I’m not sure why, staff are afraid to ask the techs to do the baths. And they need to use a little bit more of our help that we have available. But, I myself, like to do my own baths, but if I find that I’m running behind I will ask them to help out. Which you hate to do that.”  “It ultimately it falls on the RNs to at least delegate the bath so it gets done, if it hasn’t. And that’s not always the case.” |
| Decision about which soap to use |  |
|  | Q21: “I know we’re decreasing their bacterial load, and I know that a person’s body can shift their kind of bacterial load by staying in a new environment. But by knocking down whatever good bacteria are there, or the normal flora you know, normal minerals and oils that the body secretes and stuff. By removing that constantly on a daily basis, could create problems.” |
|  | Q22: “Yeah, that's kind of our, what our policy is now. We have to, within a day, 24 hours, we're supposed to try to give them a bath, chlorhexidine. We are told to, that we should be doing them. I guess. I don't always think that's the right soap to use all the time, depending, because it's very drying on the patient's skin. Or there's certain things that you shouldn't be use CHG on, and that's personal opinion. A bath should be given to the patient. What we use, I guess, it doesn't matter. You know, if they find that this is helping, fine. I mean, it all seems so more complicated. It’s just another soap.” |
|  | Q23: “I'm an old nurse, so I prefer using soap and water anyways over CHG.”  “ You know, to me it's like because in the old days it wasn't chlorhexidine, it was soap and water, and it was like these guys have been bathed, I mean, criminy, they look like little newborn babies. I haven't seen the studies, but I think soap and water is good. I mean, we use it to clean our hands. It's hard to say. You know, I mean, the other day I just gave a guy a full bath with that chlorhexidine and he still smelled. You know, he still had an odor to him. And I'm like thinking that, you know, that chlorhexidine didn't even really get rid of his body odor. You know, it was kind of strange.” |
|  | Q24: “I mean, there’s a lot of factors that would probably need to be evaluated that are different from our hospital and the ones that are actually testing this. Or actually just replicate the study in a large VA facility and see whether or not it’s actually feasible and actually seeing a significant drop.” |
| Order set intervention | Q25: “I mean that makes sense. I like that because if it’s treated as a medication then they’ll have to do it. And it'll motivate people to do it more, and not as like, they’ll treat it more as like an obligation, not like a leisure for the patient and not like a favor for the patient. They’ll treat is as an obligation of their duty, which is what they should do. |
